# A Case Report of Scrotal Squamous Cell Carcinoma Secondary to Chronic Urinary Irritation

**DOI:** 10.7759/cureus.2430

**Published:** 2018-04-05

**Authors:** Mohamed Ali Essid, Abderrazak Bouzouita, Ahmed Saadi, Ahlem Blel, Kays Chaker, Marouen Chakroun, Haroun Ayed, Mohamed Cherif, Soumaya Rammah, Mohamed Riadh Ben Slama, Amine Derouiche, Mohamed Chebil

**Affiliations:** 1 Urology, Charles Nicolle Hospital; 2 Pathology, Charles Nicolle Hospital; 3 Urology, La Rabta Hospital, Tunis, TUN

**Keywords:** scrotal cancer, squamous cell carcinoma, hypospadias

## Abstract

Most scrotum cancers are associated with occupational exposure. We report a case of a squamous cell carcinoma of the scrotum in a patient with a proximal meatus, secondary to mistreated urethral stricture. Based on our observations in this case, we think that chronic urinary inflammation of the scrotal skin may also be considered as a risk factor of scrotal cancer.

## Introduction

Scrotal cancer is very rare nowadays. In the past, occupational exposure has been associated with increased risk. With the improvement of working conditions, risk factors have changed. We present here a case report of scrotal cancer caused by urinary chronic irritation.

## Case presentation

**Figure 1:**
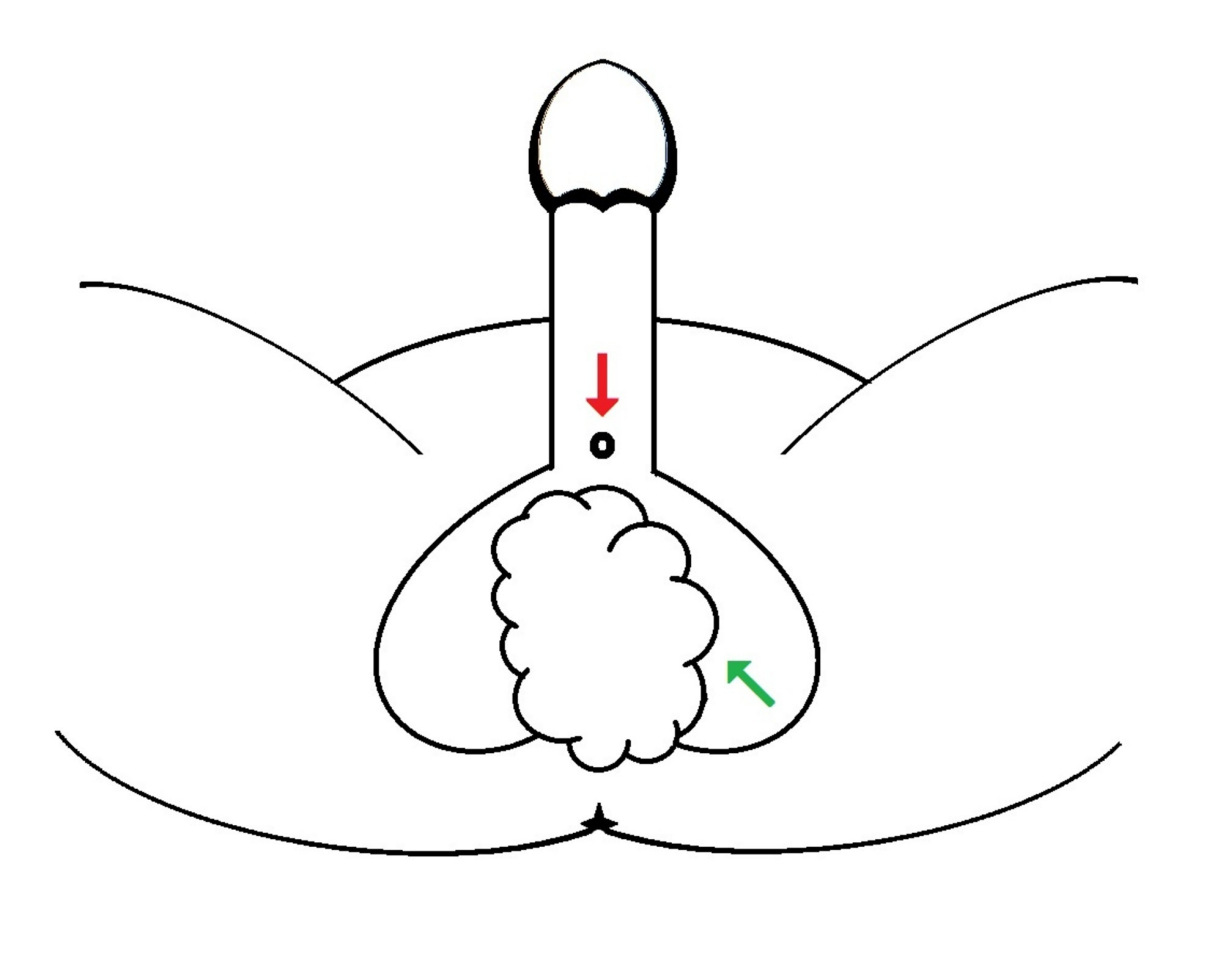
Sketch of the proximal urethral meatus (red arrow) and the scrotal lesion (green arrow) as present in our patient.

We present an 83-year-old male patient, a retired greengrocer, with a long history of anterior urethral stricture treated endoscopically in the early 90’s. Later, the patient ended up with a meatus on the penoscrotal junction. He was lost until 2015 when he consulted for a 16-month history of an ulcerated lesion of the scrotum. He urinates from the same meatus, in a sitting position, and voided urine had always wet the scrotal skin. Meanwhile, he didn’t receive further treatment. Physical examination found a 4 x 2 cm ulcero-budding lesion on the median raphe with a depth of 10–15 mm and spreading a urine odor (Figure 1).

**Figure 2:**
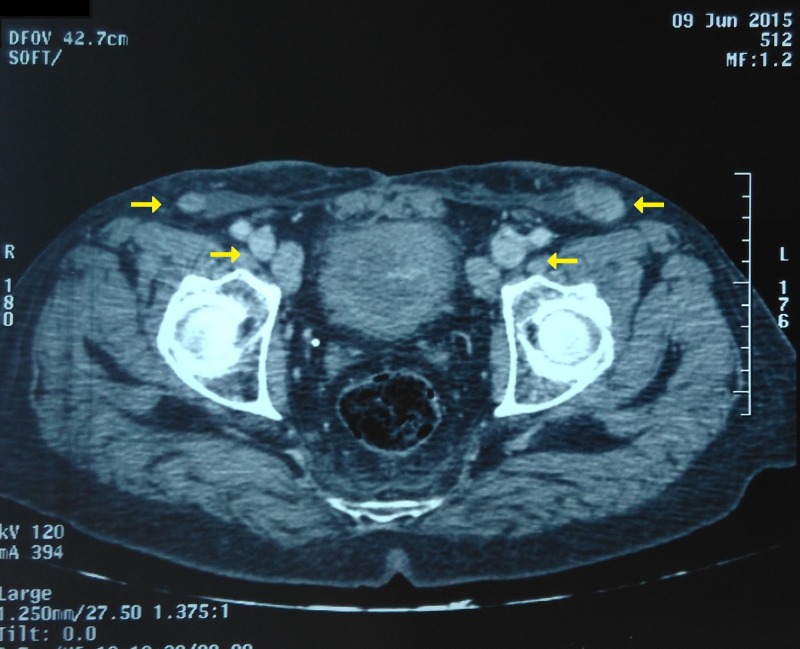
Axial section of CT scan showing bilateral iliac and inguinal lymph nodes (yellow arrows).

There were also bilateral inguinal indurate and painless nodes measuring 2–3 cm. No particularity on the testis exam and rectal touch. Apical meatus was closed. A biopsy of the lesion found a moderately differentiated squamous cell carcinoma. A thoraco-abdominopelvic computed tomography (CT) scan showed bilateral inguinal and iliac nodes and no metastasis (Figure 2).

**Figure 3:**
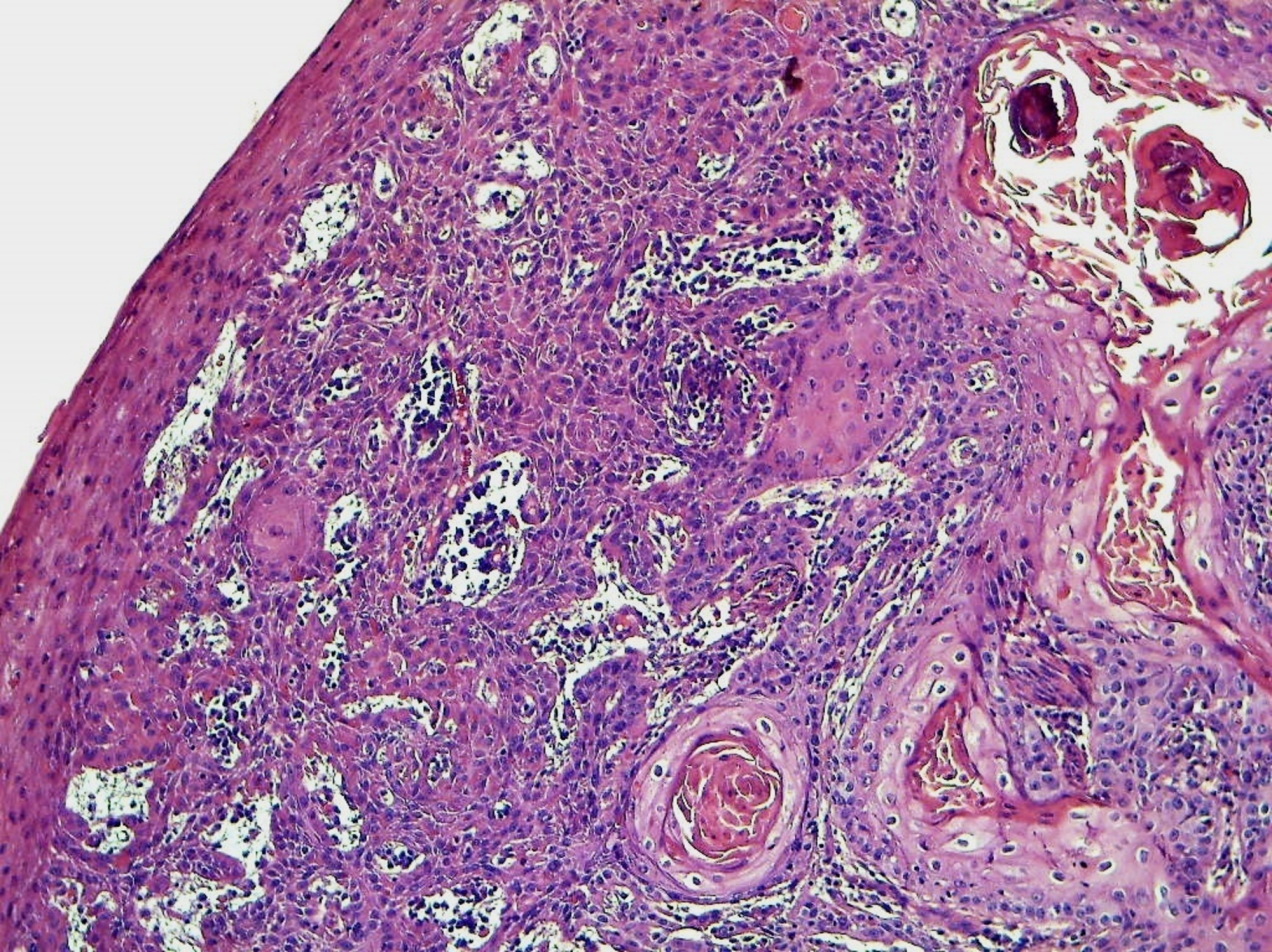
Invasive keratinizing squamous cell carcinoma developing from the epidermis (HE x 100)

The patient only underwent an incomplete excision because the tumor was locally advanced in the perineum. The penis and testis weren’t involved. Urine was drained by a cystostomy tube. Pathological examination of the surgical specimen concluded at a moderately differentiated squamous cell carcinoma (Figure 3).

Palliative radiotherapy has been discussed, but given the advanced age of the patient and his poor personal status, no further treatments were indicated. The wound took months to heal. The patient was followed regularly for 14 months until he was lost to follow up.

## Discussion

Squamous cell carcinoma (SCC) is the most common histological type of scrotal cancer. Historically, SCC was the first malignancy to be linked directly to exposure to occupational carcinogens. In addition to soot, SCC has also been linked to exposure to tar, pith, different types of lubricating and cutting oils, creosotes, gas production, and paraffin wax pressing [1].

With better occupational health practices minimizing exposure at the workplace, the disease became very rare. In the modern era, the incidence of scrotal SCC is as low as 0.35 per 1,000,000 male persons per year [2]. Most cases are thought to result from poor hygiene and chronic irritation. More precisely it can be linked to radiation history, psoriasis treatment by Psoralen-ultraviolet light, and human papillomavirus (HPV) infection [3].

The patient we report here had a more than 20 years' chronic irritation of the scrotum by urine. It seems reasonable to hypothesize that chronic contact of the scrotal skin with urine because of the meatus position resulting in chronic inflammation might have contributed to the cancer development, as described in the only similar case in the literature that occurs in a 68-year-old man with a proximal uncorrected hypospadias [4]. 

By analogy, few case reports of SCC involving the urinary stoma as ureterocutaneostomy, suprapubic cystostomy, and Mitrofanoff ureterovesical stoma were described [5]. 

## Conclusions

Through our case, we throw the light on the effect of chronic urinary irritation of the scrotum. Special attention must be paid to patients with mistreated urethral stricture or hypospadias resulting in a proximal meatus.
